# Survival for patients with metastatic colon cancer underwent cytoreductive colectomy in the era of rapid development of anticancer drugs: A real-world analysis based on updated population dataset of 2004–2018

**DOI:** 10.3389/fphar.2022.983092

**Published:** 2022-10-19

**Authors:** Guangran Meng, Shengtao Yang, Feixiang Chen

**Affiliations:** ^1^ Department of General Surgery, The Fifth Hospital of Wuhan, Wuhan, Hubei, China; ^2^ Department of Anesthesiology, The Fifth Hospital of Wuhan, Wuhan, Hubei, China

**Keywords:** survival outcomes, colon cancer, metastatic, cytoreductive colectomy, early mortality

## Abstract

**Objective:** Metastatic colon cancer (mCC) poses a great threat to the survival of patients suffering from it. In the past decade, many clinical trials have been carried out to improve the prognosis of patients with mCC. Numerous treatments have emerged, and satisfactory efficacy has been demonstrated in randomized phase III trials in highly selective patients with mCC. Our present study aims to investigate whether these therapeutic advances can be reflected to the broader mCC patients who performed cytoreductive colectomy.

**Method:** General and prognostic data for patients diagnosed with mCC who underwent cytoreductive colectomy between 2004–2018 were extracted from the Surveillance, Epidemiology, and End Results (SEER) database. Survival was analyzed using the Kaplan-Meier method and Cox proportional hazards model. The hazard ratio (HR) and its 95% confidence interval (CI) were used to evaluate the influence of risk factors on prognosis.

**Results:** A total of 26,301 patients diagnosed with mCC treated with cytoreductive colectomy were included in this study. The median overall survival was 19 months (range, 17–23). The good prognosis was associated with patients diagnosed at the most recent year, younger age, non-black race, female, married, without previous history of malignancy, no second malignancy onset, descending/sigmoid/splenic flexure colon tumor, normal CEA levels at diagnosis, low primary tumor burden, T1/T2 stage, N0 stage, single organ metastasis, underwent surgical resection of synchronous distant metastatic lymph nodes or organs, a high number of lymph-node examinations, low positive lymph-node ratio and received adjuvant chemotherapy. The proportion of patients surviving for ≥24 months increased from 37% in 2004 to 44.2% in 2016 (*p* < 0.001), especially in ≤49 years patients [46.8% in 2004 to 57.8% in 2016 (*p* < 0.001)]. The percentage of patients who died within 3 months decreased between 2004 and 2018 (from 19.6% to 15.7%; *p* < 0.001).

**Conclusion:** Over a span of 15 years, the long-term survival has improved in real-world mCC patients who were treated with cytoreductive colectomy, especially among younger patients. However, the median overall survival remains not substantial.

## Introduction

The incidence of colorectal cancer is rising in recent years, accounting for approximately 10% of all cancers. It is currently the third most common malignancy in the world, and the second most common cause of cancer-related deaths (Siegel et al., 2020; Biller and Schrag, 2021; Siegel et al., 2022). About 20% of patients with colorectal cancer are found to have distant metastases at the time of diagnosis, and up to 50% of patients progress to metastatic disease during subsequent follow-up (van der Geest et al., 2015). Metastatic colorectal cancer has a poor prognosis (Biller and Schrag, 2021; Modest et al., 2022). Since metastatic colorectal cancer encompasses a broad spectrum of clinical diseases, its clinical prognosis varies widely among patients. Unresectable metastatic disease has an extremely poor prognosis without systemic therapy and a mean OS of 6–8 months (Kawai et al., 2021). The chemotherapy regimen of 5-fluorouracil (5-FU) combined with oxaliplatin or irinotecan has been the cornerstone of the treatments of metastatic colorectal cancer since the 1960s (Douillard et al., 2000; Saltz et al., 2000; Goldberg et al., 2004; Lee et al., 2019; Modest et al., 2022). At the same time, by adding drugs that target vascular endothelial growth factor (VEGF) (such as bevacizumab, ramucirumab, and ziv-aflibercept), and drugs that inhibit the epidermal growth factor receptor (EGFR) signaling pathway (such as cetuximab and panitumumab) significantly improved median survival in patients with metastatic colorectal cancer (Mody and Bekaii-Saab, 2018). In addition, immune checkpoint inhibitors (such as pembrolizumab, and nivolumab) have made rapid progress in the field of colorectal cancer and brought new changes to the treatment of advanced colorectal cancer (Overman et al., 2017; Ganesh et al., 2019; André et al., 2020; Andre et al., 2021; Modest et al., 2022).

Through multidisciplinary comprehensive treatment strategies, the progression of colorectal cancer can be better controlled and alleviated, and the survival rate of most tumors can be improved. However, the progress made in the treatment of metastatic colorectal cancer is mostly reflected in the results of randomized controlled clinical studies, and these studies have a process of highly selective screening of cases for case inclusion. It is worth investigating whether the progress in the treatment of metastatic colorectal cancer is reflected in the real world. The purpose of this study was to investigate the short-term and long-term survival of patients with metastatic colon cancer (mCC), who underwent cytoreductive colectomy from 2004 to 2018. This will aid in determining whether the advances of metastatic colorectal cancer treatment from randomized controlled clinical studies are extended to the broader mCC population.

## Patients and methods

### Data source

The analysis data in this study were obtained from Surveillance, Epidemiology, and End Results (SEER) database (https://seer.cancer.gov/) compiled by the National Cancer Institute of the United States. The study used 18 registrations of the SEER database, which accounted for about 28% of the total population of the United States. The basic characteristics of patients were similar to those of the general population, and they were representative. Demographic information, clinical features, the incidence of cancer, and treatment and survival of patients registered by each cancer registry were recorded in the SEER database. Since the SEER database is anonymous and has nothing to do with human studies, the ethics review committee of our hospital exempted ethical approval.

### Patient identification

We identified patients with primary metastatic colon cancer, who underwent surgery from 2004 to 2018. The inclusion criteria were as follows: 1) All cases had pathological diagnosed, rather than an autopsy or death certificate. 2) Patients ≥ 18 years old. 3) Received recommended cytoreductive colectomy. 4) Complete information on causes of death and follow-up time. 5) Deleted the cases with duplicate IDs were excluded, and patients who underwent tumor reduction surgery but were not recommended by clinicians were removed.

### Study variables

Research variables included year of diagnosis (2004–2009 and 2010–2018), age of diagnosis (≤49, 50–59, 60–69, 70–79, and 80+), race (white, black, and others), gender (male and female), marital status (never married, married, and widowed/divorced/separated), history of malignant tumors (with or without), lifetime number of tumors (1, 2 and 3 and above), location of colon cancer (ascending colon, hepatic flexure, transverse colon, splenic flexure, descending colon, sigmoid colon, and large intestine NOS), tumor size (>5 cm and ≤5 cm), Carcino-embryonic antigen (CEA) level (normal, borderline, and elevated), histology (adenocarcinoma and non-adenocarcinoma), T stage (T1, T2, T3, and T4), N stage (N0, N1, and N2), M stage (M1a, M1b, M1c), metastasis site (bone, liver, lung, and brain), number of organ metastasis (1, 2, and 3+), peritoneal infiltration (yes and no), number of tumor deposits (none, 1-2, and 2+), number of lymph node examination (≤16 and >16; limited to patients with lymph node examination), number of positive lymph nodes (≤4 and >4; limited to patients with lymph node examination), lymph node positive rate (≤31% and >31%; limited to patients with lymph node examination, surgical methods (Subtotal colectomy hemicolectomy, Total colectomy/proctocolectomy, and Partial colectomy segmental/local excision), distant metastasis of lymph nodes and organs of surgery (yes and no), and radiotherapy and chemotherapy (yes and no).

### Statistical analyses

All continuous variables in this study were described as mean ± standard deviation (SD) if they conformed to a normal distribution, and were compared by Student’s *t*-test. If the variables did not conform to the normal distribution, they were described as the median and interquartile range (IQR) and compared with Wilcoxon rank-sum test. Frequency (%) was used to represent the classification variables and the chi-square test for comparison. Kaplan-Meier method was used to calculate the survival rate of patients. Univariate and multivariate Cox proportional hazard model and Fine and Gray model were used to estimate the hazard ratios (HRs) and sub-distribution hazard ratios (sHRs) of total overall survival (OS) and cancer-specific survival (CSS), and their corresponding 95% confidence intervals, respectively. This was done to assess the impact of different covariates on OS and CSS, and to determine independent risk factors. Ordered logistic regression analysis was used to evaluate the impact of different covariates on early death risk, and the odds ratio (OR) of risk factors and its 95% CI were calculated. In addition, in order to evaluate the HR of age, tumor size, tumor deposits, number of lymph node examinations, number of positive lymph nodes, and rate of lymph node-positive on OS risk, the restricted cubic spline curve was used to display these correlations. The non-linear test was carried out through the likelihood ratio test, and the log-likelihood of the model with linear term and the model with cubic spline term was compared. All *p*-values were bilateral, and a *p*-value of <0.05 was considered to be statistically significant. *R* statistical package (v. 4.2.0) was used for evaluations.

## Results

### Baseline characteristics

A total of 26,301 mCC patients were identified between 2004–2018 through the SEER database. Of these, 12,068 (45.9%) were identified between 2004–2009 and 14,233 (54.1%) between 2010–2018. Detailed demographic information and clinical characteristics are shown in Table 1 and Supplementary Table S1. The median age at diagnosis for the total population was 65 years (IQR: 54–75). The diagnostic age of patients identified between 2010–2018 [66 years (IQR: 53–74)] was less than that of those identified between 2004–2009 [64 (IQR: 55–74)]. The percentage of patients in different age groups was different before and after 2010. The proportion of patients ≤49 years increased from 14.2% to 16.8%, while the proportion of patients ≥80 years decreased from 16.8% to 14.7% from 2004–2009 to 2010–2018. The proportion of tumors >5 cm increased from 40.3% to 46.4% compares 2004–2009 with 2010–2018, while the proportion of tumors <5 cm decreased from 53.6% to 49.4%. The proportion of T3 tumors decreased from 62.8% in 2004–2009 to 52.2% in 2010–2018, while the proportion of T4 tumors increased from 33.4% to 44.5%. It was found that the number of local lymph node examinations >16 increased from 37.0% in 2004–2009 to 52.0% in 2010–2018. In addition, patients receiving chemotherapy increased from 59.2% in 2004–2009 to 67.4% in 2010–2018.

**TABLE 1:** Baseline clinicopathologic characteristics.

Characteristics	All *N* = 26,301	2004–2009 *N* = 12,068 (45.9%)	2010–2018 *N* = 14,233 (54.1%)	*p*
Age at diagnosis. Median (IQR)	65.0 (54.0; 75.0)	66.0 (55.0; 76.0)	64.0 (53.0; 74.0)	<0.001
Age at diagnosis. *n* (%)				<0.001
≤49	4,098 (15.6%)	1713 (14.2%)	2,385 (16.8%)	
50–59	5,684 (21.6%)	2,508 (20.8%)	3,176 (22.3%)	
60–69	6,710 (25.5%)	2,963 (24.6%)	3,747 (26.3%)	
70–79	5,685 (21.6%)	2,856 (23.7%)	2,829 (19.9%)	
80+	4,124 (15.7%)	2028 (16.8%)	2096 (14.7%)	
Race. *n* (%)				<0.001
White	20,025 (76.1%)	9,357 (77.5%)	10,668 (75.0%)	
Black	3,893 (14.8%)	1728 (14.3%)	2,165 (15.2%)	
Other	2,334 (8.87%)	969 (8.03%)	1,365 (9.59%)	
Missing	49 (0.19%)	14 (0.12%)	35 (0.25%)	
Sex. *n* (%)				0.061
Female	12,640 (48.1%)	5,876 (48.7%)	6,764 (47.5%)	
Male	13,661 (51.9%)	6,192 (51.3%)	7,469 (52.5%)	
Marital status. *n* (%)				<0.001
Never married	4,615 (17.5%)	1788 (14.8%)	2,827 (19.9%)	
Married	14,143 (53.8%)	6,736 (55.8%)	7,407 (52.0%)	
Widowed/divorced/separated	6,577 (25.0%)	3,208 (26.6%)	3,369 (23.7%)	
Missing	966 (3.67%)	336 (2.78%)	630 (4.43%)	
Previous tumor history. *n* (%)				0.009
No	23,115 (87.9%)	10,537 (87.3%)	12,578 (88.4%)	
Yes	3,186 (12.1%)	1,531 (12.7%)	1,655 (11.6%)	
Lifetime number of tumors. *n* (%)				<0.001
1	21,386 (81.3%)	9,650 (80.0%)	11,736 (82.5%)	
2	4,081 (15.5%)	1985 (16.4%)	2096 (14.7%)	
3+	834 (3.17%)	433 (3.59%)	401 (2.82%)	
Site of the tumor. n (%)				<0.001
Ascending colon	6,397 (24.3%)	2,855 (23.7%)	3,542 (24.9%)	
Hepatic flexure	1,616 (6.14%)	807 (6.69%)	809 (5.68%)	
Transverse colon	3,263 (12.4%)	1,503 (12.5%)	1760 (12.4%)	
Splenic flexure	1,385 (5.27%)	693 (5.74%)	692 (4.86%)	
Descending colon	2,248 (8.55%)	988 (8.19%)	1,260 (8.85%)	
Sigmoid colon	10,367 (39.4%)	4,807 (39.8%)	5,560 (39.1%)	
Large intestine, NOS	1,025 (3.90%)	415 (3.44%)	610 (4.29%)	
CEA level. *n* (%)				0.001
Normal/borderline	3,947 (15.0%)	1778 (14.7%)	2,169 (15.2%)	
Elevated	13,803 (52.5%)	6,229 (51.6%)	7,574 (53.2%)	
Missing	8,551 (32.5%)	4,061 (33.7%)	4,490 (31.5%)	
Size of tumor. Median (IQR)	5.00 (4.00; 6.50)	5.00 (3.80; 6.50)	5.00 (4.00; 6.70)	<0.001
Size of the tumor. *n* (%)				<0.001
>5 cm	11,462 (43.6%)	4,861 (40.3%)	6,601 (46.4%)	
≤5 cm	13,492 (51.3%)	6,466 (53.6%)	7,026 (49.4%)	
Missing	1,347 (5.12%)	741 (6.14%)	606 (4.26%)	
Histology. *n* (%)				<0.001
Adenocarcinoma	25,276 (96.1%)	11,661 (96.6%)	13,615 (95.7%)	
Non-adenocarcinoma	1,025 (3.90%)	407 (3.37%)	618 (4.34%)	
T stage. *n* (%)				<0.001
T1	280 (1.06%)	144 (1.19%)	136 (0.96%)	
T2	637 (2.42%)	301 (2.49%)	336 (2.36%)	
T3	15,010 (57.1%)	7,578 (62.8%)	7,432 (52.2%)	
T4	10,374 (39.4%)	4,045 (33.5%)	6,329 (44.5%)	
*N* stage. *n* (%)				<0.001
N0	4,795 (18.2%)	2,321 (19.2%)	2,474 (17.4%)	
N1	9,088 (34.6%)	3,934 (32.6%)	5,154 (36.2%)	
N2	12,418 (47.2%)	5,813 (48.2%)	6,605 (46.4%)	
M stage. *n* (%)				
M1a	8,215 (31.2%)		8,215 (57.7%)	
M1b	5,098 (19.4%)		5,098 (35.8%)	
M1c	273 (1.04%)		273 (1.92%)	
M1, NOS	12,715 (48.3%)		647 (4.55%)	
Bone metastasis. *n* (%)				
No	13,591 (51.7%)		13,591 (95.5%)	
Bone	372 (1.41%)		372 (2.61%)	
Missing	12,338 (46.9%)		270 (1.90%)	
Brain metastasis. *n* (%)				
No	13,821 (52.5%)		13,821 (97.1%)	
Brain	120 (0.46%)		120 (0.84%)	
Missing	12,360 (47.0%)		292 (2.05%)	
Liver metastasis. *n* (%)				
No	4,042 (15.4%)		4,042 (28.4%)	
Liver	10,055 (38.2%)		10,055 (70.6%)	
Missing	12,204 (46.4%)		136 (0.96%)	
Lung metastasis. *n* (%)				
No	11,777 (44.8%)		11,777 (82.7%)	
Lung	2,176 (8.27%)		2,176 (15.3%)	
Missing	12,348 (46.9%)		280 (1.97%)	
Metastasis site. *n* (%)				
Liver	8,511 (32.4%)		8,511 (59.8%)	
Liver + lung	1,266 (4.81%)		1,266 (8.89%)	
Lung	746 (2.84%)		746 (5.24%)	
Bone/brain only or combine with other	476 (1.81%)		476 (3.34%)	
Other	3,065 (11.7%)		3,065 (21.5%)	
Missing	12,237 (46.5%)		169 (1.19%)	
Number of metastasis. *n* (%)				
1	9,392 (35.7%)		9,392 (66.0%)	
2	1,494 (5.68%)		1,494 (10.5%)	
3+	113 (0.43%)		113 (0.79%)	
Other	3,065 (11.7%)		3,065 (21.5%)	
Missing	12,237 (46.5%)		169 (1.19%)	
Perineural invasion. *n* (%)				
No	8,640 (32.9%)		8,640 (60.7%)	
Yes	4,113 (15.6%)		4,113 (28.9%)	
Missing	13,548 (51.5%)		1,480 (10.4%)	
Number of tumor deposits. Median (IQR)	2.00 (1.00; 5.00)		2.00 (1.00; 5.00)	
The number of tumor deposits. *n* (%)				
≤2	1,294 (4.92%)		1,294 (9.09%)	
>2	1,244 (4.73%)		1,244 (8.74%)	
None	8,061 (30.6%)		8,061 (56.6%)	
NA	15,702 (59.7%)		3,634 (25.5%)	
Regional lymphnodes examined. Median (IQR)	16.0 (11.0; 22.0)	14.0 (9.00; 20.0)	17.0 (13.0; 23.0)	<0.001
Regional lymphnodes were examined. *n* (%)				<0.001
≤16	14,424 (54.8%)	7,598 (63.0%)	6,826 (48.0%)	
>16	11,877 (45.2%)	4,470 (37.0%)	7,407 (52.0%)	
Regional lymphnodes positive. Median (IQR)	4.00 (2.00; 8.00)	4.00 (2.00; 8.00)	4.00 (2.00; 8.00)	0.006
Regional lymphnodes positive. *n* (%)				<0.001
≤4	11,008 (41.9%)	4,915 (40.7%)	6,093 (42.8%)	
>4	10,498 (39.9%)	4,832 (40.0%)	5,666 (39.8%)	
No	4,795 (18.2%)	2,321 (19.2%)	2,474 (17.4%)	
Rate of regional lymphnodes positive. Median (IQR)	0.31 [0.14; 0.59]	0.36 [0.17; 0.67]	0.27 [0.12; 0.52]	<0.001
Rate of regional lymphnodes positive. *n* (%)				<0.001
≤31%	10,765 (40.9%)	4,258 (35.3%)	6,507 (45.7%)	
>31%	10,741 (40.8%)	5,489 (45.5%)	5,252 (36.9%)	
No	4,795 (18.2%)	2,321 (19.2%)	2,474 (17.4%)	
Surgery type. *n* (%)				0.017
Subtotal colectomy/hemicolectomy	13,262 (50.4%)	6,013 (49.8%)	7,249 (50.9%)	
Total colectomy/proctocolectomy	783 (2.98%)	330 (2.73%)	453 (3.18%)	
Partial colectomy/segmental/local excision	11,706 (44.5%)	5,458 (45.2%)	6,248 (43.9%)	
Colectomy, NOS	550 (2.09%)	267 (2.21%)	283 (1.99%)	
Surgery for distant metastasis organ and lymphnode site. *n* (%)				<0.001
No	19,140 (72.8%)	9,012 (74.7%)	10,128 (71.2%)	
Yes	7,121 (27.1%)	3,033 (25.1%)	4,088 (28.7%)	
Missing	40 (0.15%)	23 (0.19%)	17 (0.12%)	
Radiation therapy. *n* (%)				0.737
No/missing	25,482 (96.9%)	11,687 (96.8%)	13,795 (96.9%)	
Yes	819 (3.11%)	381 (3.16%)	438 (3.08%)	
Chemotherapy therapy. *n* (%)				<0.001
No/missing	9,565 (36.4%)	4,924 (40.8%)	4,641 (32.6%)	
Yes	16,736 (63.6%)	7,144 (59.2%)	9,592 (67.4%)	

IQR: interquartile range; CEA: Carcino-embryonic antigen.

### Survival outcomes

**FIGURE 1:**
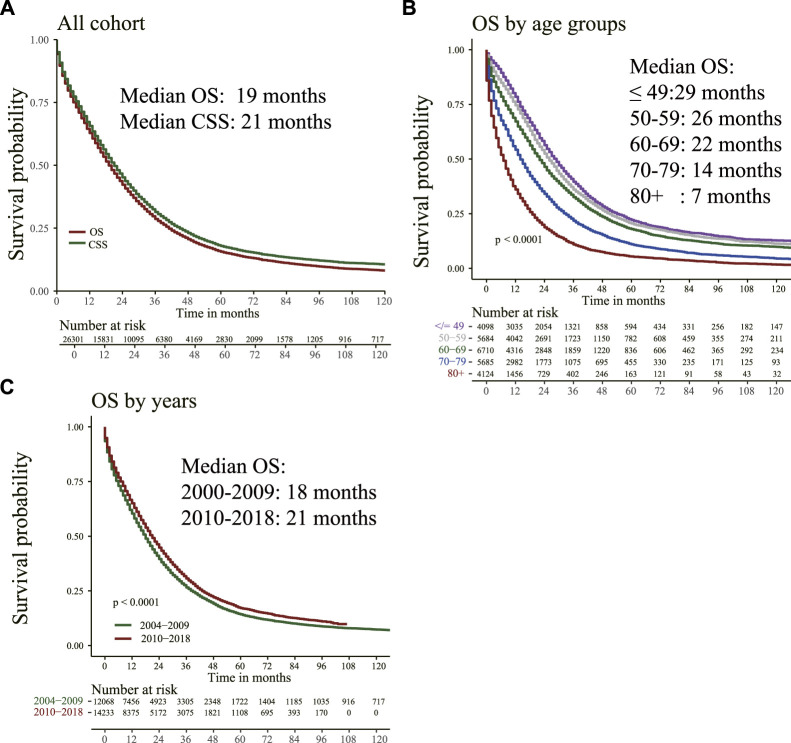
Overall survival (OS) and cancer-specific survival (CSS) were estimated by Kaplan–Meier analysis. **(A)** OS and CSS curve; **(B)** OS in different age groups; **(C)** OS of patients diagnosed at different time points.

Figure 1 shows that for the total population, the median OS is 19 months, and the median CSS is 21 months. For patients in different age groups, older patients had shorter median OS. For instance, patients ≤49 years had a median OS of 29 months, whereas for those >80 years was 7 months. The median OS of patients with mCC from 2004–2009 was 18 months, and for patients from 2010–2018 was 21 months.

The HR of death risk showed a consistent trend of statistically significant improvement after 2010. The HR of death risk of patients diagnosed at 2018 was 0.62 (95% CI, 0.50–0.77; *p* < 0.001) compared to patients diagnosed at 2004 (Table 2).

**TABLE 2:** Univariate analysis of hazard ratio for mortality.

	Median OS	HR (95%CI)	*p*	Adjusted HR (95%CI) ^a^	*p*
Year at diagnosis. *n* (%)					
2004	17	1 reference		1 reference	
2005	17	1.01 (0.95–1.08)	0.741	1.01 (0.95–1.07)	0.808
2006	18	0.99 (0.93–1.06)	0.866	1.03 (0.96–1.09)	0.429
2007	19	0.95 (0.89–1.01)	0.106	0.99 (0.93–1.06)	0.853
2008	18	0.98 (0.92–1.04)	0.545	1.04 (0.98–1.11)	0.206
2009	19	0.94 (0.88–1.00)	0.062	1.01 (0.94–1.07)	0.880
2010	20	0.92 (0.87–0.99)	0.017	0.82 (0.68–0.98)	0.027
2011	19	0.93 (0.87–0.99)	0.024	0.85 (0.71–1.01)	0.070
2012	20	0.89 (0.83–0.95)	0.001	0.79 (0.66–0.95)	0.011
2013	20	0.87 (0.81–0.94)	<0.001	0.80 (0.67–0.97)	0.019
2014	23	0.83 (0.77–0.89)	<0.001	0.78 (0.65–0.93)	0.007
2015	23	0.82 (0.76–0.88)	<0.001	0.74 (0.62–0.89)	0.002
2016	21	0.82 (0.76–0.89)	<0.001	0.73 (0.60–0.88)	0.001
2017	21	0.82 (0.75–0.90)	<0.001	0.73 (0.60–0.89)	0.002
2018	NA	0.68 (0.60–0.77)	<0.001	0.62 (0.50–0.77)	<0.001

^a^ Adjusted for the covariables of age at diagnosis, race, sex, marital status, previous tumor history, lifetime number of tumors, site of the tumor, CEA, level, size of the tumor, histology, T and N stage, regional lymphnodes examined, surgery type, surgery for distant metastasis organ and lymphnode site, radiation therapy, and chemotherapy therapy.

HR: hazard ratio; CI: confidence interval; OS: Overall survival.

**FIGURE 2:**
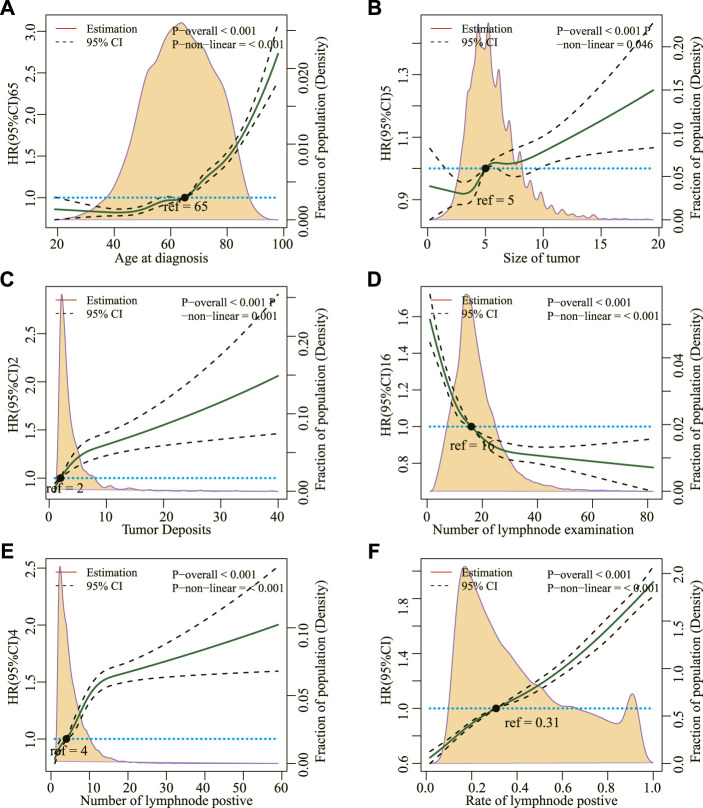
**(A)** Restricted cubic splines for the association between hazard risk of overall mortality and age at diagnosis, **(B)** Size of tumor, **(C)** Tumor Deposits, **(D)** Number of lymph node examination, **(E)** Number of lymph node positives, and **(F)** Rate of lymph node positives. Solid lines represent hazard ratio (HR); dashed lines represent 95% CIs. The hazard risk estimates were adjusted for all other covariables.

Figure 2 shows that age, tumor size, tumor deposits, number of regional lymph node tests, number of regional lymph node positives, and positive rate of regional lymph nodes at the time of diagnosis did not linearly correlate with the risk of all-cause deaths in patients with mCC (*p* for non-linear <0.05). These factors were then transformed into categorical variables and incorporated into the Cox model. After adjusting other covariates, they were all found to be independent risk factors for all-cause deaths (Table 3).

**TABLE 3:** Median overall survival and 2-year OS, and analyses of variables associated with OS.

	Survival function^a^		Cox model analysis			
Characteristics	Median OS (95%CI)	2-year OS(95%CI)	HR (95%CI) #	*P*	Adjusted HR (95%CI)	*P*
Year at diagnosis						
2004–2009	18 (17–18)	40.0% (39.0%–40.0%)	1 reference		1 reference	
2010–2018	21 (20–22)	45.0% (44.0%–46.0%)	0.88 (0.86–0.91)	<0.001	0.79 (0.67–0.94)^b^	0.007
Age at diagnosis						
≤49	29 (27–30)	56.0% (55.0%–58.0%)	1 reference		1 reference	
50–59	26 (25–27)	53.0% (51.0%–54.0%)	1.09 (1.04–1.14)	<0.001	1.05 (1.00–1.10)^b^	0.035
60–69	22 (21–23)	46.0% (45.0%–48.0%)	1.24 (1.19–1.30)	<0.001	1.16 (1.11–1.21)^b^	<0.001
70–79	14 (14–15)	34.0% (33.0%–35.0%)	1.73 (1.65–1.81)	<0.001	1.47 (1.40–1.54)^b^	<0.001
80+	7 (7–8)	19.0% (18.0%–20.0%)	2.70 (2.57–2.84)	<0.001	1.91 (1.81–2.01)^b^	<0.001
Race						
White	19 (18–19)	42.0% (41.0%–42.0%)	1 reference		1 reference	
Black	20 (19–20)	41.0% (40.0%–43.0%)	1.04 (1.00–1.08)	0.034	1.06 (1.02–1.10)^b^	0.006
Other	23 (22–25)	48.0% (46.0%–50.0%)	0.85 (0.81–0.90)	<0.001	0.90 (0.86–0.95)^b^	0.000
Sex						
Female	19 (18–19)	41.0% (40.0%–42.0%)	1 reference		1 reference	
Male	20 (20–20)	43.0% (42.0%–44.0%)	1.00 (0.97–1.02)	0.828	1.05 (1.02–1.08)^b^	0.002
Marital status						
Never married	20 (19–21]	43.0% (42.0%–45.0%)	1 reference		1 reference	
Married	22 (21–22)	46.0% (45.0%–47.0%)	0.93 (0.90–0.97)	<0.001	0.91 (0.87–0.94)^b^	<0.001
Widowed/Divorced/Separated	14 (14–15)	34.0% (33.0%–35.0%)	1.24 (1.19–1.29)	<0.001	0.99 (0.95–1.04)^b^	0.783
Previous tumor history						
No	20 (20–21)	44.0% (43.0%–44.0%)	1 reference		1 reference	
Yes	14 (13–14)	32.0% (31.0%–34.0%)	1.31 (1.26–1.37)	<0.001	1.65 (1.54–1.76)^b^	<0.001
Lifetime number of tumors						
1	20 (19–20)	42.0% (42.0%–43.0%)	1 reference		1 reference	
2	18 (17–19)	41.0% (40.0%–43.0%)	1.00 (0.96–1.04)	0.991	0.67 (0.63–0.71)^b^	<0.001
3+	19 (17–22)	43.0% (40.0%–47.0%)	0.94 (0.87–1.01)	0.092	0.54 (0.49–0.59)^b^	<0.001
Site of tumor						
Ascending colon	14 (14–15)	32.0% (31.0%–33.0%)	1 reference		1 reference	
Hepatic flexure	14 (13–16)	33.0% (31.0%–35.0%)	0.98 (0.92–1.04)	0.426	1.04 (0.98–1.10)^b^	0.193
Transverse colon	16 (15–17)	36.0% (34.0%–38.0%)	0.93 (0.88–0.97)	0.001	0.98 90.94–1.03)^b^	0.427
Splenic flexure	21 (20–23)	45.0% (43.0%–48.0%)	0.75 (0.71–0.80)	<0.001	0.85 (0.79–0.91)^b^	<0.001
Descending colon	23 (22–25)	48.0% (46.0%–51.0%)	0.73 (0.69–0.77)	<0.001	0.84 (0.79–0.89)^b^	<0.001
Sigmoid colon	25 (25–26)	51.0% (50.0%–52.0%)	0.69 (0.67–0.72)	<0.001	0.80 (0.76–0.83)^b^	<0.001
Large intestine, NOS	13 (11–15)	31.0% (29.0%–35.0%)	1.09 (1.01–1.17)	0.026	1.04 (0.97–1.12)^b^	0.313
CEA level						
Normal/borderline	27 (25–28)	53.0% (51.0%–54.0%)	1 reference		1 reference	
Elevated	19 (19–20)	41.0% (41.0%–42.0%)	1.43 (1.38–1.49)	<0.001	1.44 (1.38–1.50)^b^	<0.001
Size of tumor						
>5 cm	17 (17–18)	39.0% (38.0%–40.0%)	1 reference		1 reference	
≤5 cm	21 (21–22)	45.0% (44.0%–46.0%)	0.89 (0.86–0.91)	<0.001	0.90 (0.88–0.93)^b^	<0.001
Histology						
Adenocarcinoma	20 (20–20)	43.0% (43.0%–44.0%)	1 reference		1 reference	
Non-adenocarcinoma	7 (6–8)	21.0% (18.0%–23.0%)	1.79 (1.67–1.91)	<0.001	1.53 (1.42–1.63)^b^	<0.001
T stage						
T1	35 (30–41)	62.0% (57.0%–68.0%)	1 reference		1 reference	
T2	30 (27–34)	57.0% (53.0%–61.0%)	1.07 (0.91–1.27)	0.420	0.97 (0.82–1.15)^b^	0.712
T3	22 (22–23)	47.0% (46.0%–47.0%)	1.42 (1.23–1.63)	<0.001	1.20 (1.05–1.39)^b^	0.010
T4	15 (15–16)	34.0% (33.0%–35.0%)	1.89 (1.64–2.17)	<0.001	1.60 (1.39–1.85)^b^	<0.001
N stage						
N0	28 (27–29)	55.0% (53.0%–56.0%)	1 reference		1 reference	
N1	22 (21–23)	46.0% (45.0%–47.0%)	1.25 (1.20–1.30)	<0.001	1.36 (1.31–1.42)^b^	<0.001
N2	16 (15–16)	35.0% (34.0%–35.0%)	1.68 (1.62–1.74)	<0.001	1.93 (1.85–2.01)^b^	<0.001
Metastasis site						
Liver	23 (22–23)	47.0% (46.0%–48.0%)	1 reference		1 reference	
Liver + lung	15 (14–17)	32.0% (29.0%–35.0%)	1.52 (1.42–1.63)	<0.001	1.09 (0.85–1.40)^b^	0.503
Lung	25 (24–28)	52.0% (48.0%–56.0%)	0.90 (0.82–0.99)	0.023	0.87 (0.79–0.96)^b^	0.003
Bone/brain only or combine with other	7 (6–9)	19.0% (16.0%–24.0%)	1.99 (1.80–2.21)	<0.001	1.36 (1.11–1.66)^b^	0.003
Other	22 (20–23)	46.0% (44.0%–48.0%)	0.99 (0.94–1.04)	0.605	0.82 (0.78–0.86)^b^	<0.001
Number of metastasis						
1	23 (22–23)	47.0% (46.0%–48.0%)	1 reference		1 reference	
2	14 (13–15)	30.0% (27.0%–32.0%)	1.61 (1.51–1.71)	<0.001	1.35 (1.06–1.72)^b^	0.015
3+	7 (6–12)	15.0% (9.0%–24.0%)	2.28 (1.87–2.78)	<0.001	1.52 (1.15–2.01)^b^	0.003
Other	22 (20–23)	46.0% (44.0%–48.0%)	0.99 (0.94–1.04)	0.694	NA	NA
Perineural invasion						
No	22 (22–23)	47.0% (46.0%–48.0%)	1 reference		1 reference	
Yes	19 (18–20)	41.0% (39.0%–43.0%)	1.20 (1.15–1.25)	<0.001	1.11 (1.06–1.16)^e^	<0.001
Number of tumor deposits						
≤2	21 (20–23)	45.0% (42.0%–48.0%)	1 reference		1 reference	
>2	16 (14–17)	36.0% (33.0%–39.0%)	1.27 (1.16–1.40)	<0.001	1.20 (1.09–1.32)^e^	0.000
None	25 (24–26)	50.0% (49.0%–51.0%)	0.87 (0.80–0.93)	<0.001	0.90 (0.84–0.97)^e^	0.007
Regional lymphnodes examined						
≤16	17 (17–18)	38.0% (38.0%–39.0%)	1 reference		1 reference	
>16	22 (22–23)	47.0% (46.0%–48.0%)	0.79 (0.77–0.81)	<0.001	0.78 (0.75–0.80)^b^	<0.001
Regional lymphnodes positive						
≤4	22 (22–23)	46.0% (45.0%–47.0%)	1 reference		1 reference	
>4	15 (14–15)	32.0% (32.0%–33.0%)	1.41 (1.37–1.45)	<0.001	1.47 (1.43–1.52)^d^	<0.001
No	28 (27–29)	55.0% (53.0%–56.0%)	0.79 (0.76–0.83)	<0.001	0.72 (0.69–0.75)^d^	<0.001
Rate of regional lymphnodes positive						
≤31%	24 (24–25)	50.0% (49.0%–51.0%)	1 reference		1 reference	
>31%	13 (13–13)	30.0% (29.0%–31.0%)	1.65 (1.60–1.70)	<0.001	1.58 (1.53–1.63)^c^	<0.001
No	28 (27–29)	55.0% (53.0%–56.0%)	0.86 (0.82–0.89)	<0.001	0.76 (0.73–0.79)^c^	<0.001
Surgery type						
Subtotal colectomy/hemicolectomy	17 (16–17)	38.0% (37.0%–39.0%)	1 reference		1 reference	
Total colectomy/proctocolectomy	18 (15–19)	38.0% (34.0%–41.0%)	1.02 (0.94–1.11)	0.574	1.20 (1.10–1.30)^b^	<0.001
Partial colectomy/segmental/Local excision	23 (22–23)	47.0% (46.0%–48.0%)	0.84 (0.82–0.87)	<0.001	0.99 (0.96–1.02)^b^	0.523
Colectomy, NOS	24 (21–26)	48.0% (43.0%–52.0%)	0.79 (0.72–0.87)	<0.001	0.88 (0.80–0.97)^b^	0.010
Surgery for distant metastasis organ and lymphnode site						
No	17 (17–18)	39.0% (38.0%–39.0%)	1 reference		1 reference	
Yes	26 (25–27)	52.0% (51.0%–53.0%)	0.71 (0.69–0.74)	<0.001	0.81 (0.79–0.84)^b^	<0.001
Radiation therapy						
No/missing	19 (19–20)	42.0% (42.0%–43.0%)	1 reference		1 reference	
Yes	19 (17–21)	42.0% (39.0%–46.0%)	0.96 (0.88–1.03)	0.261	1.05 (0.97–1.14)^b^	0.202
Chemotherapy therapy						
No/missing	6 (6–6)	21.0% (20.0%–22.0%)	1 reference		1 reference	
Yes	27 (27–28)	54.0% (54.0%–55.0%)	0.41 (0.40–0.43)	<0.001	0.46 (0.45–0.47)^b^	<0.001

OS, overall survival; HR, hazard ratio; CEA, carcino-embryonic antigen; CI, confidence interval; NA, not applicable.

^a^ Based on the overall dataset presented in Table 1, and the missing variables were not present here since these variables have no clinical significance.

^b^ All variables are included in one Cox model.

^c^ Adjusted for the covariables of age at diagnosis, race, sex, marital status, previous tumor history, lifetime number of tumors, site of the tumor, CEA, level, size of the tumor, histology, T stage, regional lymphnodes examined, surgery type, surgery for distant metastasis organ and lymphnode site, radiation therapy, and chemotherapy therapy.

^d^ Adjusted for the covariables of age at diagnosis, race, sex, marital status, previous tumor history, lifetime number of tumors, site of the tumor, CEA, level, size of the tumor, histology, T stage, regional lymphnodes examined, surgery type, surgery for distant metastasis organ and lymphnode site, radiation therapy, and chemotherapy therapy.

^e^ Adjusted for the covariables of age at diagnosis, race, sex, marital status, previous tumor history, lifetime number of tumors, site of the tumor, CEA, level, size of the tumor, histology, T and N stage, regional lymphnodes examined, surgery type, surgery for distant metastasis organ and lymphnode site, radiation therapy, and chemotherapy therapy based on the dataset of 2010–2018 which included the information of perineural invasion and number of tumor deposits.

### Short- and long-term survivors

**FIGURE 3:**
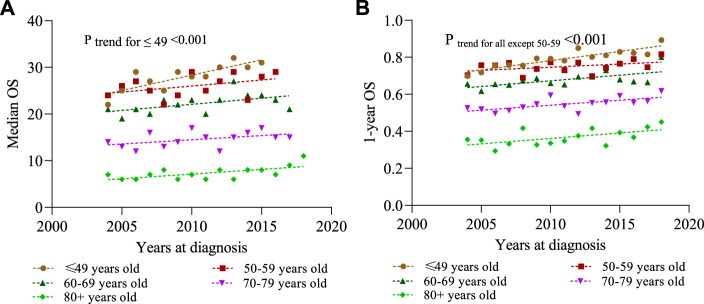
Overall survival (OS) is estimated by Kaplan–Meier method for different years at diagnosis stratified by different ages in the diagnosis cohort. **(A)** Median OS; **(B)** 1-year OS.

**FIGURE 4:**
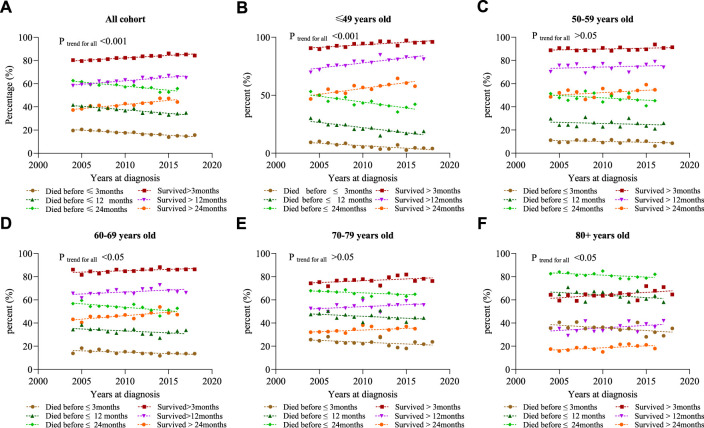
Percentage of death events in different years at diagnosis. **(A)** All cohort; **(B)** ≤ 49 years; **(C)** 50–59 years; **(D)** 60–69 years; **(E)** 70–79 years; and **(F)** 80 +years.

We define short-term survivors as patients who died within 3 months of initial diagnosis, and long-term survivors as patients who survived for at least 24 months. Figure 4 shows that the proportion of short-term survivors in the total population decreased from 19.6% in 2004 to 13.9% in 2015 (*p* < 0.001). The proportion of long-term survivors increased from 62.7% in 2004 to 55.8% in 2016 (*p* < 0.001). In addition, survivors who survived for at least 12 months increased from 58.4% in 2004 to 67.0% in 2015. It was found that the trend of survival improvement was more prominent in the younger population (Figures 3, 4).

The good long-term survival was associated with patients diagnosed at the most recent year, younger age, non-black race, female, married, without previous history of malignancy, no second malignancy onset, descending/sigmoid/splenic flexure colon tumor, normal CEA levels at diagnosis, low primary tumor burden, T1/T2 stage, N0 stage, single organ metastasis, underwent surgical resection of synchronous distant metastatic lymph nodes or organs, a high number of lymph-node examinations, low positive lymph-node ratio and received adjuvant chemotherapy (Table 4).

**TABLE 4:** Factors associated with early death in ordered logistic regression^a^.

	Baseline clinicopathologic characteristics of patients dead in the different time interval						Multivariable model	
	≤ 3 month *N* = 4,564 (21.5%)	4–12 months *N* = 4,958 (23.4%)	13–24 months *N* = 4,904 (23.1%)	25–36 months *N* = 3,029 (14.3%)	>36 months *N* = 3,753 (17.7%)	*p*.trend	Adjusted OR (95%CI)	*p*
Age at diagnosis. Median (IQR)	74.0 (63.0; 82.0)	69.0 (58.0; 78.8)	63.0 (54.0; 73.2)	61.0 (52.0; 71.0)	61.0 (52.0; 70.0)	<0.001		
Age at diagnosis. *n* (%)						<0.001		
≤49	249 (5.46%)	598 (12.1%)	805 (16.4%)	565 (18.7%)	728 (19.4%)		1 reference	
50–59	565 (12.4%)	837 (16.9%)	1,134 (23.1%)	792 (26.1%)	950 (25.3%)		1.10 (1.01–1.19)^b^	0.018
60–69	975 (21.4%)	1,169 (23.6%)	1,267 (25.8%)	790 (26.1%)	1,080 (28.8%)		1.26 (1.16–1.37)^b^	<0.001
70–79	1,316 (28.8%)	1,229 (24.8%)	1,045 (21.3%)	601 (19.8%)	711 (18.9%)		1.74 (1.59–1.90)^b^	<0.001
80+	1,459 (32.0%)	1,125 (22.7%)	653 (13.3%)	281 (9.28%)	284 (7.57%)		2.24 (2.02–2.47)^b^	<0.001
Race. *n* (%)						<0.001		
White	3,609 (79.1%)	3,819 (77.0%)	3,695 (75.3%)	2,255 (74.4%)	2,862 (76.3%)		1 reference	
Black	650 (14.2%)	724 (14.6%)	793 (16.2%)	485 (16.0%)	567 (15.1%)		0.97 (0.90–1.04)^b^	0.193
Other	300 (6.57%)	412 (8.31%)	411 (8.38%)	286 (9.44%)	320 (8.53%)		0.90 (0.82–0.98)^b^	0.008
Missing	5 (0.11%)	3 (0.06%)	5 (0.10%)	3 (0.10%)	4 (0.11%)		0.61 (0.27–1.39)^b^	0.115
Sex. *n* (%)						<0.001		
Female	2,296 (50.3%)	2,463 (49.7%)	2,312 (47.1%)	1,337 (44.1%)	1729 (46.1%)		1 reference	
Male	2,268 (49.7%)	2,495 (50.3%)	2,592 (52.9%)	1,692 (55.9%)	2024 (53.9%)		0.95 (0.90–0.99)^b^	0.029
Marital status. *n* (%)						<0.001		
Never married	757 (16.6%)	846 (17.1%)	846 (17.3%)	543 (17.9%)	586 (15.6%)		1 reference	
Married	2041 (44.7%)	2,579 (52.0%)	2,675 (54.5%)	1736 (57.3%)	2,252 (60.0%)		0.87 (0.81–0.94)^b^	<0.001
Widowed/divorced/separated	1,591 (34.9%)	1,353 (27.3%)	1,206 (24.6%)	657 (21.7%)	796 (21.2%)		0.99 (0.91–1.07)^b^	0.406
Missing	175 (3.83%)	180 (3.63%)	177 (3.61%)	93 (3.07%)	119 (3.17%)		0.98 (0.85–1.14)^b^	0.393
Previous tumor history. *n* (%)						<0.001		
No	3,805 (83.4%)	4,222 (85.2%)	4,329 (88.3%)	2,727 (90.0%)	3,379 (90.0%)		1 reference	
Yes	759 (16.6%)	736 (14.8%)	575 (11.7%)	302 (9.97%)	374 (9.97%)		2.11 (1.86–2.39)^b^	<0.001
Lifetime number of tumors. *n* (%)						0.131		
1	3,604 (79.0%)	3,965 (80.0%)	4,080 (83.2%)	2,512 (82.9%)	2,973 (79.2%)		1 reference	
2	790 (17.3%)	838 (16.9%)	689 (14.0%)	439 (14.5%)	627 (16.7%)		0.55 (0.50–0.61)^b^	<0.001
3+	170 (3.72%)	155 (3.13%)	135 (2.75%)	78 (2.58%)	153 (4.08%)		0.41 (0.34–0.49)^b^	<0.001
Site of the tumor. *n* (%)						<0.001		
Ascending colon	1,420 (31.1%)	1,455 (29.3%)	1,269 (25.9%)	573 (18.9%)	657 (17.5%)		1 reference	
Hepatic flexure	317 (6.95%)	389 (7.85%)	327 (6.67%)	160 (5.28%)	158 (4.21%)		1.03 (0.92–1.15)^b^	0.301
Transverse colon	712 (15.6%)	682 (13.8%)	609 (12.4%)	326 (10.8%)	377 (10.0%)		1.00 (0.91–1.09)^b^	0.466
Splenic flexure	234 (5.13%)	230 (4.64%)	259 (5.28%)	166 (5.48%)	202 (5.38%)		0.82 (0.72–0.92)^b^	<0.001
Descending colon	327 (7.16%)	397 (8.01%)	376 (7.67%)	296 (9.77%)	374 (9.97%)		0.71 (0.64–0.78)^b^	<0.001
Sigmoid colon	1,296 (28.4%)	1,576 (31.8%)	1884 (38.4%)	1,404 (46.4%)	1894 (50.5%)		0.65 (0.60–0.70)^b^	<0.001
Large intestine, NOS	258 (5.65%)	229 (4.62%)	180 (3.67%)	104 (3.43%)	91 (2.42%)		0.96 (0.84–1.10)^b^	0.296
CEA level. *n* (%)						<0.001		
Normal/borderline	462 (10.1%)	638 (12.9%)	661 (13.5%)	420 (13.9%)	640 (17.1%)		1 reference	
Elevated	2,317 (50.8%)	2,671 (53.9%)	2,664 (54.3%)	1,667 (55.0%)	1913 (51.0%)		1.43 (1.33–1.55)^b^	<0.001
Missing	1785 (39.1%)	1,649 (33.3%)	1,579 (32.2%)	942 (31.1%)	1,200 (32.0%)		1.24 (1.15–1.35)^b^	<0.001
Size of tumor. Median (IQR)	5.30 (4.00; 7.00)	5.00 (4.00; 7.00)	5.00 (4.00; 6.50)	5.00 (3.80; 6.40)	4.70 (3.50; 6.00)	<0.001		
Size of the tumor. *n* (%)						<0.001		
>5 cm	2,207 (48.4%)	2,356 (47.5%)	2089 (42.6%)	1,206 (39.8%)	1,398 (37.3%)		1 reference	
≤5 cm	2,118 (46.4%)	2,371 (47.8%)	2,586 (52.7%)	1,676 (55.3%)	2,138 (57.0%)		0.81 (0.77–0.85)^b^	<0.001
NA	239 (5.24%)	231 (4.66%)	229 (4.67%)	147 (4.85%)	217 (5.78%)		0.97 (0.86–1.09)^b^	0.289
Histology. *n* (%)						<0.001		
Adenocarcinoma	4,204 (92.1%)	4,680 (94.4%)	4,760 (97.1%)	2,964 (97.9%)	3,701 (98.6%)		1 reference	
Non-adenocarcinoma	360 (7.89%)	278 (5.61%)	144 (2.94%)	65 (2.15%)	52 (1.39%)		2.24 (1.97–2.55)^b^	<0.001
T stage. *n* (%)						<0.001		
T1	40 (0.88%)	27 (0.54%)	34 (0.69%)	33 (1.09%)	66 (1.76%)		1 reference	
T2	79 (1.73%)	82 (1.65%)	96 (1.96%)	72 (2.38%)	122 (3.25%)		1.04 (0.76–1.43)^b^	0.401
T3	2,222 (48.7%)	2,598 (52.4%)	2,829 (57.7%)	1879 (62.0%)	2,473 (65.9%)		1.20 (0.92–1.57)^b^	0.092
T4	2,223 (48.7%)	2,251 (45.4%)	1945 (39.7%)	1,045 (34.5%)	1,092 (29.1%)		1.81 (1.38–2.36)^b^	<0.001
N stage. *n* (%)						<0.001		
N0	647 (14.2%)	693 (14.0%)	720 (14.7%)	558 (18.4%)	900 (24.0%)		1 reference	
N1	1,376 (30.1%)	1,550 (31.3%)	1,691 (34.5%)	1,079 (35.6%)	1,423 (37.9%)		1.10 (0.92–1.33)^b^	0.151
N2	2,541 (55.7%)	2,715 (54.8%)	2,493 (50.8%)	1,392 (46.0%)	1,430 (38.1%)		1.43 (1.19–1.73)^b^	<0.001
Metastasis site. *n* (%)						<0.001		
Liver	1,247 (27.3%)	1,395 (28.1%)	1,449 (29.5%)	923 (30.5%)	911 (24.3%)		1 reference	
Liver + lung	260 (5.70%)	269 (5.43%)	249 (5.08%)	135 (4.46%)	78 (2.08%)		1.54 (1.36–1.74^b^	<0.001
Lung	93 (2.04%)	133 (2.68%)	102 (2.08%)	75 (2.48%)	82 (2.18%)		0.78 (0.66–0.92)^b^	0.002
Bone/brain only or combine with other	151 (3.31%)	133 (2.68%)	75 (1.53%)	28 (0.92%)	10 (0.27%)		2.22 (1.83–2.69)^b^	<0.001
Other	451 (9.88%)	551 (11.1%)	498 (10.2%)	298 (9.84%)	281 (7.49%)		0.82 (0.75–0.90)^b^	<0.001
Missing	2,362 (51.8%)	2,477 (50.0%)	2,531 (51.6%)	1,570 (51.8%)	2,391 (63.7%)		0.77 (0.56–1.05)^b^	0.051
The number of metastasis. *n* (%)						<0.001		
1	1,380 (30.2%)	1,563 (31.5%)	1,566 (31.9%)	1,005 (33.2%)	996 (26.5%)		1 reference	
2	336 (7.36%)	335 (6.76%)	287 (5.85%)	149 (4.92%)	82 (2.18%)		1.69 (1.52–1.90)^c^	<0.001
3+	35 (0.77%)	32 (0.65%)	22 (0.45%)	7 (0.23%)	3 (0.08%)		2.76 (1.93–3.97)^c^	<0.001
Other	451 (9.88%)	551 (11.1%)	498 (10.2%)	298 (9.84%)	281 (7.49%)		0.84 (0.76–0.92)^c^	<0.001
Missing	2,362 (51.8%)	2,477 (50.0%)	2,531 (51.6%)	1,570 (51.8%)	2,391 (63.7%)		0.82 (0.78–0.87)^c^	<0.001
Perineural invasion. *n* (%)						<0.001		
No	1,258 (27.6%)	1,484 (29.9%)	1,424 (29.0%)	901 (29.7%)	862 (23.0%)		1 reference	
Yes	670 (14.7%)	764 (15.4%)	741 (15.1%)	432 (14.3%)	353 (9.41%)		1.08 (0.98–1.20)^d^	0.062
Missing	2,636 (57.8%)	2,710 (54.7%)	2,739 (55.9%)	1,696 (56.0%)	2,538 (67.6%)			
Number of tumor deposits. Median (IQR)	3.00 (1.00; 6.00)	3.00 (2.00; 7.00)	2.00 (1.00; 5.00)	2.00 (1.00; 4.00)	2.00 (1.00; 4.00)	<0.001		
The number of tumor deposits. *n* (%)						<0.001		
≤2	178 (3.90%)	205 (4.13%)	221 (4.51%)	127 (4.19%)	90 (2.40%)		1 reference	
>2	225 (4.93%)	272 (5.49%)	200 (4.08%)	111 (3.66%)	58 (1.55%)		1.18 (0.99–1.42)^d^	0.037
No	1,074 (23.5%)	1,276 (25.7%)	1,310 (26.7%)	869 (28.7%)	891 (23.7%)		0.88 (0.76–1.01)^d^	0.035
NA	3,087 (67.6%)	3,205 (64.6%)	3,173 (64.7%)	1922 (63.5%)	2,714 (72.3%)			
Regional lymphnodes examined. Median (IQR)	14.0 (9.00; 19.0)	15.0 (11.0; 21.0)	15.0 (11.0; 21.0)	16.0 (12.0; 21.0)	15.0 (11.0; 21.0)	<0.001		
Regional lymphnodes were examined. *n* (%)						<0.001		
≤16	2,952 (64.7%)	2,855 (57.6%)	2,756 (56.2%)	1,652 (54.5%)	2084 (55.5%)		1 reference	
>16	1,612 (35.3%)	2,103 (42.4%)	2,148 (43.8%)	1,377 (45.5%)	1,669 (44.5%)		0.70 (0.67–0.74)^a^	<0.001
Regional lymphnodes positive. Median (IQR)	6.00 (3.00; 10.0)	5.00 (3.00; 10.0)	5.00 (2.00; 8.00)	4.00 (2.00; 7.00)	4.00 (2.00; 6.00)	<0.001		
Regional lymphnodes positive. *n* (%)						0.482		
≤4	1,636 (35.8%)	1902 (38.4%)	2057 (41.9%)	1,329 (43.9%)	1729 (46.1%)		1 reference	
>4	2,281 (50.0%)	2,363 (47.7%)	2,127 (43.4%)	1,142 (37.7%)	1,124 (29.9%)		1.65 (1.56–1.74)^c^	<0.001
No	647 (14.2%)	693 (14.0%)	720 (14.7%)	558 (18.4%)	900 (24.0%)		0.72 (0.67–0.78)^c^	<0.001
Rate of regional lymphnodes positive. Median (IQR)	0.48 (0.22; 0.80)	0.39 (0.19; 0.70)	0.33 (0.15; 0.60)	0.28 (0.13; 0.50)	0.25 (0.12; 0.44)	<0.001		
Rate of regional lymphnodes positive. *n* (%)						<0.001		
≤31%	1,338 (29.3%)	1739 (35.1%)	2001 (40.8%)	1,346 (44.4%)	1714 (45.7%)		1 reference	
>31%	2,579 (56.5%)	2,526 (50.9%)	2,183 (44.5%)	1,125 (37.1%)	1,139 (30.3%)		1.87 (1.77–1.98)^c^	<0.001
No	647 (14.2%)	693 (14.0%)	720 (14.7%)	558 (18.4%)	900 (24.0%)		0.80 (0.74–0.86)^c^	<0.001
Surgery type. *n* (%)						<0.001		
Subtotal colectomy/hemicolectomy	2,570 (56.3%)	2,752 (55.5%)	2,517 (51.3%)	1,372 (45.3%)	1,610 (42.9%)		1 reference	
Total colectomy/proctocolectomy	151 (3.31%)	168 (3.39%)	144 (2.94%)	84 (2.77%)	99 (2.64%)		1.23 (1.06–1.42)^a^	0.004
Partial colectomy/segmental/Local excision	1762 (38.6%)	1942 (39.2%)	2,146 (43.8%)	1,501 (49.6%)	1966 (52.4%)		0.88 (0.83–0.93)^a^	<0.001
Colectomy, NOS	81 (1.77%)	96 (1.94%)	97 (1.98%)	72 (2.38%)	78 (2.08%)		0.84 (0.70–1.01)^a^	0.029
Surgery for distant metastasis organ and lymphnode site. *n* (%)						<0.001		
No	3,632 (79.6%)	3,875 (78.2%)	3,682 (75.1%)	2,215 (73.1%)	2,493 (66.4%)		1 reference	
Yes	929 (20.4%)	1,075 (21.7%)	1,211 (24.7%)	810 (26.7%)	1,257 (33.5%)		0.78 (0.73–0.82)^a^	<0.001
Missing	3 (0.07%)	8 (0.16%)	11 (0.22%)	4 (0.13%)	3 (0.08%)		0.92 (0.48–1.75)^a^	0.398
Radiation therapy. *n* (%)						0.04		
No/missing	4,474 (98.0%)	4,767 (96.1%)	4,741 (96.7%)	2,940 (97.1%)	3,631 (96.7%)		1 reference	
Yes	90 (1.97%)	191 (3.85%)	163 (3.32%)	89 (2.94%)	122 (3.25%)		1.24 (1.08–1.43)^a^	0.001
Chemotherapy therapy. *n* (%)						<0.001		
No/missing	3,927 (86.0%)	2,111 (42.6%)	1,291 (26.3%)	597 (19.7%)	728 (19.4%)		1 reference	
Yes	637 (14.0%)	2,847 (57.4%)	3,613 (73.7%)	2,432 (80.3%)	3,025 (80.6%)		0.17 (0.16–0.19)^a^	<0.001

CEA, carcino-embryonic antigen; OR, odds ratio; CI, confidence interval.

^a^ Odds ratio in ordered logistic regression is interpreted as the increased odds of death with early time interval.

^b^ All variables are included in the ordered logistic regression model.

^c^ Respectively adjusted for the covariables of age at diagnosis, race, sex, marital status, previous tumor history, lifetime number of tumors, site of the tumor, CEA, level, size of the tumor, histology, T stage, regional lymphnodes examined, surgery type, surgery for distant metastasis organ and lymphnode site, radiation therapy, and chemotherapy therapy.

^d^ Respectively adjusted for the covariables of age at diagnosis, race, sex, marital status, previous tumor history, lifetime number of tumors, site of the tumor, CEA, level, size of the tumor, histology, T and N stage, regional lymphnodes examined, surgery type, surgery for distant metastasis organ and lymphnode site, radiation therapy, and chemotherapy therapy based on the dataset of 2010–2018 which included the information of perineural invasion and number of tumor deposits.

## Discussion

The study mainly found that the median OS of patients with mCC, who underwent cytoreductive surgery, remained largely stable and not substantial (17–21 months) over a duration of 15 years, however, the risk of mortality for mCC patients who underwent cytoreductive surgery has decreased in recent years compared to the past. Besides, we focusing on patients at both extremes of the survival spectrum and found that the percentage of patients who died within 3 months decreased and the percentage of patients who survival more than 24 months increased, which fully affirms the survival benefits of colon cancer patients underwent cytoreductive surgery in the era of rapid development of anticancer drugs.

Since the 1990s, 5-FU in combination with leucovorin has become the mainstay of treatment for metastatic colorectal cancer, and studies have reported a median survival of approximately 12 months (Poon et al., 1989). Subsequent studies have evaluated the effects of FOLFOX (a combination of 5-FU, leucovorin, and oxaliplatin) and FOLFIRI (a combination of 5-FU, leucovorin, and irinotecan) for metastatic colorectal cancer. These multidrug regimens resulted in a median OS of 12–20 months (de Gramont et al., 2000; Douillard et al., 2000; Saltz et al., 2000), suggesting that the multidrug combination may prolong patient survival. Therefore, FOLFOX and FOLFIRI regimens have become the standard first-line chemotherapy regimens for the treatment of metastatic colorectal cancer, and there is no significant difference in tumor benefit between the two regimens (Lee et al., 2019).

The emergence of molecularly targeted drugs has further improved the treatment of metastatic colorectal cancer. Bevacizumab, a monoclonal antibody targeting VEGF, inhibits tumor angiogenesis in combination with chemotherapy. Hurwitz et al. (2004) showed that compared with FOLFIRI alone, FOLFIRI combined with bevacizumab significantly prolonged the OS of patients (15.6 months vs. 20.3 months). The use of FOLFOXIRI combined with bevacizumab in patients with unresectable metastatic colorectal cancer was shown to have a significant overall objective response rate (69%), with a respectable conversion rate of 40% in selected patients (Tomasello et al., 2017). Other targeted angiogenesis drugs, such as ziv-aflibercept and ramucirumab, are recommended in combination with FOLFIRI for second-line treatment of metastatic colorectal cancer, and their median progression-free survival and objective response rate are superior to FOLFIRI therapy alone (Lee et al., 2019). Another important target for metastatic colorectal cancer therapy is EGFR, a tyrosine kinase closely related to HER2/neu that is overexpressed in many tumors. It is involved in signaling pathways, such as tumor proliferation, angiogenesis, and migration. Cetuximab and panitumumab are EGFR inhibitors and were found to have a significant survival advantage over supportive care in patients with chemotherapy-refractory tumors, and when combined with chemotherapy as first-line therapy, it significantly improved PFS and OS (Riedesser et al., 2022). Currently, anti-EGFR therapy is mainly used for treating metastatic colorectal cancer patients with wild-type RAS/BRAF, since RAS/BRAF mutant patients do not respond well to anti-EGFR therapy.

The RAS gene is often mutated in metastatic colorectal cancer, and the most mutated gene is the Kirsten Ras (KRAS) gene. More than 40% of metastatic colorectal cancers have KRAS mutations (Ros et al., 2021; Ciardiello et al., 2022). RAS mutations are often associated with poor prognosis and decreased response to antitumor therapy. Therefore, treatments targeting this RAS gene mutation are beneficial for the majority of patients. Currently, there is a lack of effective drugs for the treatment of RAS gene mutations. Sotorasib and adagrasib selectively inhibit KRAS^G12C^ mutation. Preliminary research results confirm their efficacy in KRAS^G12C^ mutation patients, and follow-up research is still in progress (Hong et al., 2020; Weiss et al., 2021). BRAF mutations are present in approximately 10–15% of patients with metastatic colorectal cancer. The V600E mutation is the most common mutation in the BRAF gene. BRAF mutations abnormally activate the MAPK signaling pathway, making tumors highly aggressive. Patients with BRAF V600E-mutated metastatic colorectal cancer are poorly responsive to chemotherapy and have an extremely poor prognosis with a median survival of only 12 months (Cohen et al., 2021). Some selective BRAF inhibitors have been developed, such as vemurafenib and dabrafenib. Unfortunately, these BRAF inhibitors negatively activate the MAPK signaling pathway, making their single-agent efficacy less than ideal. Combining multi-target blockade may help to obtain a more effective antitumor response. The BEACON study confirms that encorafenib plus cetuximab has significant advantages in objective response rate and OS in BRAF V600E-mutated metastatic colorectal cancer (Tabernero et al., 2021) In addition, studies combining encorafenib, cetuximab, and chemotherapy are also underway (Kopetz et al., 2021). Aberrant alterations in the HER2 gene are relatively uncommon in metastatic colorectal cancer, with HER2 gene amplification present in approximately 3% of metastatic colorectal cancers, mostly in RAS/BRAF wild-type patients (Ross et al., 2018). Retrospective studies have shown that HER2 gene amplification is associated with resistance to anti-EFGR therapy (Raghav et al., 2019). Currently, a series of studies on anti-HER2 therapy drugs in RAS/BRAF WT metastatic colorectal cancer patients is underway, and encouraging results have been obtained. This indicates that targeting HER2 therapy has a strong potential for treating metastatic colorectal cancer (Meric-Bernstam et al., 2019; Sartore-Bianchi et al., 2020; Siena et al., 2021).

Immunotherapy has yielded favorable outcomes in patients with a variety of solid tumors. However, in metastatic colorectal cancer, only a small proportion of patients may benefit. Microsatellite-instability-high (MSI-H)/mismatch repair-deficient (dMMR) is a biomarker for predicting the efficacy of immunotherapy in metastatic colorectal cancer, and it is present in approximately 5% of tumors in metastatic colorectal cancer (Arrichiello et al., 2021). Results of a multicenter randomized phase III clinical trial showed that compared with chemotherapy, pembrolizumab significantly prolonged median progression-free survival (PFS) in dMMR/MSI-H colorectal cancer patients, reaching 16.5 months. The majority of pembrolizumab monoclonal antibody-treated patients achieved objective responses over time (84% of patients lasting ≥2 years) (André et al., 2020). For microsatellite stable (MSS)/mismatch repair proficient (pMMR) patients with a high proportion of metastatic colorectal cancer, the efficacy of immunotherapy is poor, and whether it is suitable to receive immunotherapy is still under investigation. Studies have shown that cytotoxic drugs, anti-angiogenic drugs, molecularly targeted therapy, and radiotherapy can activate immunogenic cell death in tumor cells. Therefore, a series of studies are investigating the potential role of immune checkpoint inhibitor combination therapy (Ciardiello et al., 2022).

Reduction in the early death of cancer patients is an important indicator to evaluate the effect of comprehensive cancer treatment and nursing. McPhail et al. (2015) analyzed early mortality in patients with breast, colorectal, lung, prostate, and ovarian cancer in the United Kingdom, and found that age, tumor stage at diagnosis, income, and geographic location were significantly associated with early mortality in colorectal cancer. An analysis of colorectal cancer patients in the United Kingdom between 2006 and 2008 found that around 11.5% of colon cancer patients died within a month of being diagnosed, and about 33% died within a year of being diagnosed. Of these, old age, late tumor stage, poverty, and visits to the emergency department were associated with early mortality (Downing et al., 2013). Lieu et al. (2014) found that age was significantly associated with OS within 1 year of diagnosis in patients with metastatic colorectal cancer. The age effect was U-shaped, wherein both, young and old age, were unfavorable factors for early mortality in patients with metastatic colorectal cancer. Younger patients are generally healthier and have fewer underlying diseases than older patients, but their OS within 1 year of diagnosis is suboptimal, which may suggest different tumor biology. Renfro et al. (2016) found that low BMI was associated with an increased risk of metastatic colorectal cancer progression and death. Metastatic colorectal cancer patients are prone to cachexia, which leads to a significant decrease in BMI, affects the subsequent treatment of patients, and results in a significant increase in the risk of death.

A pooled analysis of 9 clinical trials examined the impact of performance status (PS) on chemotherapy in patients with metastatic colorectal cancer (Sargent et al., 2009). Although patients with PS grade 2 achieved similar treatment benefits as those with PS grades 0 and 1, they had significantly higher 60-day mortality (12.0% vs. 2.8%, *p* < 0.001). This suggests that performance status is an important risk factor for early mortality in patients with metastatic colorectal cancer. In a phase III randomized controlled trial of irinotecan in metastatic colorectal cancer, Giessen et al. (2011) assessed the clinicopathological factors associated with 60-day mortality in patients. The 60-day mortality rate in the study was 5.0% (24/479). Elevated LDH and WBC levels were considered independent predictors for early mortality, and fitness status showed a negative trend with an increased risk of early mortality in the study, but it was not statistically significant. However, in another study by Giessen et al. (2013). WBC count and performance status were identified as significant risk factors for early mortality, whereas LDH levels were not found to be associated with early mortality. In a randomized controlled study of primary tumor resection combined with systemic therapy (van der Kruijssen et al., 2021), patients with metastatic colorectal cancer who received systemic therapy after primary tumor resection had significantly higher 60-day mortality than patients who received systemic therapy alone. In the surgical group, factors such as serum lactate dehydrogenase, aspartate aminotransferase, alanine aminotransferase, and neutrophil count were associated with 60-day mortality.

In addition, the 60-day mortality appears to be significantly high in patients with multiple risk factors before surgery. There may be multiple reasons for how these factors affect early mortality in patients. 1) These risk factors are considered indicators of tumor mutational burden and malignancy (Kleespies et al., 2009; Ahn et al., 2014; Turner et al., 2015). Elevated levels of multiple indicators in patients suggest a large tumor burden, and therefore patients are at a risk of rapid progression after surgery. 2) These biochemical markers have been associated with poor prognosis in multiple studies (Ahn et al., 2014; Li et al., 2016). 3) Elevated levels of neutrophils in patients suggest a more pronounced systemic inflammatory response, which significantly increases the mortality of patients (Dell'Aquila et al., 2018). Early mortality was analyzed in a pooled analysis of 28 randomized clinical trials of metastatic colorectal cancer, which collected data from more than 22,000 patients with metastatic colorectal cancer from the ARCAD database. In this analysis, early mortality at 30 days, 60 days, and 90 days was 1.4%, 3.4%, and 5.5%, respectively. Older age, lower BMI, poor performance status, multiple metastatic sites, BRAF mutation status, and elevated laboratory markers (elevated bilirubin, WBC count, and neutrophil count) are associated with 90-day death. In contrast, KRAS mutation status, sex, single metastases, primary tumor site, and prior chemotherapy and treatment class (targeted versus non-targeted) were not associated with early mortality (Renfro et al., 2017). For patients with metastatic colorectal cancer with multiple risk factors, treatment options should be carefully evaluated to improve patient survival.

Our study has several limitations. First, the study was retrospective and the analysis relied on administrative claims data, with the potential for misclassification of cancer stage, vital status, and cause-specific survival. Second, the research object of this article is patients with mCC who underwent cytoreductive colectomy, it had clumped together a very heterogenous disease into a single category, which may be misleading. For example, the cohort will include patients with oligometastatic disease who underwent curative surgery and patients with bowel obstruction who underwent surgery, one has good outcomes, while the other is of poor prognosis. While, considering the different severity of metastatic colon cancer corresponding to different cytoreductive colectomy type and had a different survival outcome, we had classified the types of cytoreductive colectomy (Subtotal colectomy/hemicolectomy, Total colectomy/proctocolectomy, Partial colectomy/segmental/Local excision) and included into multivariate analysis. These type of cytoreductive colectomy are common in clinical practice for metastatic colon cancer patients. Third, there is no information on patient Karnofsky performance status, comorbidities, drug use of chemotherapy and targeted immunotherapy, or information on gene mutation, and imaging information (computerized tomography, magnetic resonance imaging, Positron Emission Tomography-Computed Tomography and so on) in the SEER database. However, the SEER database has done the following to ensure data accuracy: population-based case identification, detailed review of medical and pathological records, strict data collection and quality control standards, and high patient follow-up rates. This data has been thoroughly audited for accuracy and completeness. Therefore, it is reasonable to assume that our data on patient characteristics, tumor pathology and staging, treatment modalities, and survival status are reasonably accurate, and even with limited data, our conclusions remain reasonable. In addition, it should be noted that this study mainly included metastatic colon cancer patients who had undergone cytoreductive surgery and did not study rectal cancer, so the conclusions are not applicable to the metastatic rectal cancer population.

## Conclusion

Over a span of 15 years, the OS and long-term survival of patients with mCC improved slightly, especially in the younger patient population. This demonstrates the progress in the comprehensive treatment for mCC over the past few decades. At the same time, we need to recognize that there is still a lot of room for improvement in the future.

## Data Availability

The original contributions presented in the study are included in the article/Supplementary Material, further inquiries can be directed to the corresponding author.
