# Supplementary material for: Survival for patients with metastatic colon cancer underwent cytoreductive colectomy in the era of rapid development of anticancer drugs: A real-world analysis based on updated population dataset of 2004–2018
Source: Front Pharmacol. 2022 Oct 19;13:983092. doi: 10.3389/fphar.2022.983092 (PMC9627288; doi:10.3389/fphar.2022.983092)
Supplement: Supplementary file 1 [file DataSheet1.pdf]

Table S1. Baseline clinicopathologic characteristics by surgery type

	Subtotal colectomy/hemicolectomy N=13262	Total colectomy/proctocolectomy N=783	Partial colectomy/segmental/Local excision N=11706	Colectomy, NOS N=550	P.overall	P.trend
**Year at diagnosis. median [IQR]**					0.017	0.022
2000-2009	6013 (45.3%)	330 (42.1%)	5458 (46.6%)	267 (48.5%)		
2010-2018	7249 (54.7%)	453 (57.9%)	6248 (53.4%)	283 (51.5%)		
**Age at diagnosis. median [IQR]**	66.0 [56.0;76.0]	62.0 [52.0;72.5]	63.0 [53.0;74.0]	60.0 [50.0;70.0]	<0.001	<0.001
**Age at diagnosis. n (%)**					<0.001	<0.001
≤ 49	1806 (13.6%)	167 (21.3%)	1996 (17.1%)	129 (23.5%)		
50-59	2700 (20.4%)	166 (21.2%)	2682 (22.9%)	136 (24.7%)		
60-69	3337 (25.2%)	209 (26.7%)	3032 (25.9%)	132 (24.0%)		
70-79	3056 (23.0%)	133 (17.0%)	2397 (20.5%)	99 (18.0%)		
80+	2363 (17.8%)	108 (13.8%)	1599 (13.7%)	54 (9.82%)		
**Race. n (%)**					.	0.13
White	9987 (75.3%)	601 (76.8%)	9027 (77.1%)	410 (74.5%)		
Black	2239 (16.9%)	133 (17.0%)	1444 (12.3%)	77 (14.0%)		
Other	1011 (7.62%)	48 (6.13%)	1214 (10.4%)	61 (11.1%)		
Missing	25 (0.19%)	1 (0.13%)	21 (0.18%)	2 (0.36%)		
**Sex. n (%)**					<0.001	0.007
Female	6552 (49.4%)	359 (45.8%)	5348 (45.7%)	381 (69.3%)		
Male	6710 (50.6%)	424 (54.2%)	6358 (54.3%)	169 (30.7%)		
**Marital status. n (%)**					0.001	0.004
Never married	2324 (17.5%)	149 (19.0%)	2031 (17.4%)	111 (20.2%)		
Married	6989 (52.7%)	408 (52.1%)	6457 (55.2%)	289 (52.5%)		
Widowed/Divorced/Separated	3434 (25.9%)	204 (26.1%)	2814 (24.0%)	125 (22.7%)		
NA	515 (3.88%)	22 (2.81%)	404 (3.45%)	25 (4.55%)		
**Previous tumor history. n (%)**					0.001	0.001
No	11579 (87.3%)	669 (85.4%)	10369 (88.6%)	498 (90.5%)		
Yes	1683 (12.7%)	114 (14.6%)	1337 (11.4%)	52 (9.45%)		
**Lifetime number of tumors. n (%)**					<0.001	<0.001
1	10650 (80.3%)	566 (72.3%)	9710 (82.9%)	460 (83.6%)		
2	2183 (16.5%)	160 (20.4%)	1664 (14.2%)	74 (13.5%)		
3+	429 (3.23%)	57 (7.28%)	332 (2.84%)	16 (2.91%)		
**Site of the tumor. n (%)**					<0.001	<0.001
Ascending Colon	5385 (40.6%)	133 (17.0%)	794 (6.78%)	85 (15.5%)		
Hepatic Flexure	1357 (10.2%)	37 (4.73%)	201 (1.72%)	21 (3.82%)		
Transverse Colon	1839 (13.9%)	109 (13.9%)	1247 (10.7%)	68 (12.4%)		
Splenic Flexure	734 (5.53%)	43 (5.49%)	586 (5.01%)	22 (4.00%)		
Descending Colon	1403 (10.6%)	83 (10.6%)	727 (6.21%)	35 (6.36%)		
Sigmoid Colon	1860 (14.0%)	322 (41.1%)	7899 (67.5%)	286 (52.0%)		
Large Intestine, NOS	684 (5.16%)	56 (7.15%)	252 (2.15%)	33 (6.00%)		
**CEA level. n (%)**					<0.001	0.046
Normal/borderline	2136 (16.1%)	122 (15.6%)	1611 (13.8%)	78 (14.2%)		
Elevated	6766 (51.0%)	396 (50.6%)	6373 (54.4%)	268 (48.7%)		
Missing	4360 (32.9%)	265 (33.8%)	3722 (31.8%)	204 (37.1%)		

**Size of tumor. median [IQR]**	5.00 [4.00;7.00]	5.00 [4.00;7.00]	5.00 [3.70;6.00]	5.40 [4.00;7.00]	<0.001	<0.001
**Size of the tumor. n (%)**					<0.001	<0.001
> 5 cm	6246 (47.1%)	355 (45.3%)	4601 (39.3%)	260 (47.3%)		
≤ 5 cm	6410 (48.3%)	358 (45.7%)	6487 (55.4%)	237 (43.1%)		
Missing	606 (4.57%)	70 (8.94%)	618 (5.28%)	53 (9.64%)		
**Histology. n (%)**					<0.001	<0.001
Adenocarcinoma	12647 (95.4%)	733 (93.6%)	11380 (97.2%)	516 (93.8%)		
Non-adenocarcinoma	615 (4.64%)	50 (6.39%)	326 (2.78%)	34 (6.18%)		
**T stage. n (%)**					<0.001	0.473
T1	121 (0.91%)	19 (2.43%)	133 (1.14%)	7 (1.27%)		
T2	316 (2.38%)	15 (1.92%)	299 (2.55%)	7 (1.27%)		
T3	7555 (57.0%)	378 (48.3%)	6899 (58.9%)	178 (32.4%)		
T4	5270 (39.7%)	371 (47.4%)	4375 (37.4%)	358 (65.1%)		
**N stage. n (%)**					<0.001	<0.001
N0	2227 (16.8%)	159 (20.3%)	2298 (19.6%)	111 (20.2%)		
N1	4508 (34.0%)	276 (35.2%)	4111 (35.1%)	193 (35.1%)		
N2	6527 (49.2%)	348 (44.4%)	5297 (45.3%)	246 (44.7%)		
**M stage. n (%)**					<0.001	0.616
M1a	4093 (30.9%)	215 (27.5%)	3796 (32.4%)	111 (20.2%)		
M1b	2636 (19.9%)	204 (26.1%)	2111 (18.0%)	147 (26.7%)		
M1c	153 (1.15%)	10 (1.28%)	100 (0.85%)	10 (1.82%)		
nM1	6380 (48.1%)	354 (45.2%)	5699 (48.7%)	282 (51.3%)		
**Bone metastasis. n (%)**					0.024	0.008
No	6941 (52.3%)	437 (55.8%)	5945 (50.8%)	268 (48.7%)		
Bone	179 (1.35%)	11 (1.40%)	176 (1.50%)	6 (1.09%)		
Missing	6142 (46.3%)	335 (42.8%)	5585 (47.7%)	276 (50.2%)		
**Brain metastasis. n (%)**					.	0.009
1No	7048 (53.1%)	444 (56.7%)	6060 (51.8%)	269 (48.9%)		
Brain	65 (0.49%)	3 (0.38%)	49 (0.42%)	3 (0.55%)		
Missing	6149 (46.4%)	336 (42.9%)	5597 (47.8%)	278 (50.5%)		
**Liver metastasis. n (%)**					<0.001	<0.001
No	2175 (16.4%)	164 (20.9%)	1559 (13.3%)	144 (26.2%)		
Liver	5009 (37.8%)	287 (36.7%)	4626 (39.5%)	133 (24.2%)		
Missing	6078 (45.8%)	332 (42.4%)	5521 (47.2%)	273 (49.6%)		
**Lung metastasis. n (%)**					<0.001	0.002
No	6061 (45.7%)	388 (49.6%)	5094 (43.5%)	234 (42.5%)		
Lung	1051 (7.92%)	58 (7.41%)	1027 (8.77%)	40 (7.27%)		
Missing	6150 (46.4%)	337 (43.0%)	5585 (47.7%)	276 (50.2%)		
**Metastasis Site. n (%)**					<0.001	0.874
Liver	4266 (32.2%)	245 (31.3%)	3892 (33.2%)	108 (19.6%)		
Liver + lung	617 (4.65%)	33 (4.21%)	597 (5.10%)	19 (3.45%)		
Lung	364 (2.74%)	22 (2.81%)	342 (2.92%)	18 (3.27%)		
Bone/brain only or combine with other	235 (1.77%)	13 (1.66%)	219 (1.87%)	9 (1.64%)		
Other	1685 (12.7%)	138 (17.6%)	1120 (9.57%)	122 (22.2%)		
Missing	6095 (46.0%)	332 (42.4%)	5536 (47.3%)	274 (49.8%)		
**The number of metastasis. n (%)**					.	0.764
1	4708 (35.5%)	271 (34.6%)	4285 (36.6%)	128 (23.3%)		

2	727 (5.48%)	38 (4.85%)	705 (6.02%)	24 (4.36%)		
3+	47 (0.35%)	4 (0.51%)	60 (0.51%)	2 (0.36%)		
Other	1685 (12.7%)	138 (17.6%)	1120 (9.57%)	122 (22.2%)		
Missing	6095 (46.0%)	332 (42.4%)	5536 (47.3%)	274 (49.8%)		
**Perineural invasion. n (%)**					0.005	0.038
No	4443 (33.5%)	272 (34.7%)	3764 (32.2%)	161 (29.3%)		
Yes	2009 (15.1%)	134 (17.1%)	1895 (16.2%)	75 (13.6%)		
Missing	6810 (51.3%)	377 (48.1%)	6047 (51.7%)	314 (57.1%)		
**Number of tumor deposits. median [IQR]**	2.00 [1.00;5.00]	2.00 [1.00;6.00]	2.00 [1.00;5.00]	3.00 [1.75;5.00]	0.693	0.628
**The number of tumor deposits. n (%)**					0.012	0.07
≤ 2	674 (5.08%)	45 (5.75%)	555 (4.74%)	20 (3.64%)		
> 2	647 (4.88%)	37 (4.73%)	532 (4.54%)	28 (5.09%)		
No	4042 (30.5%)	242 (30.9%)	3646 (31.1%)	131 (23.8%)		
NA	7899 (59.6%)	459 (58.6%)	6973 (59.6%)	371 (67.5%)		
**Regional lymphnodes examined. median [IQR]**	17.0 [12.0;23.0]	18.0 [13.0;27.0]	14.0 [10.0;20.0]	15.0 [10.0;22.0]	<0.001	<0.001
**Regional lymphnodes were examined. n (%)**					<0.001	<0.001
≤ 16	6619 (49.9%)	326 (41.6%)	7170 (61.3%)	309 (56.2%)		
> 16	6643 (50.1%)	457 (58.4%)	4536 (38.7%)	241 (43.8%)		
**Regional lymphnodes positive. median [IQR]**	5.00 [2.00;9.00]	4.00 [2.00;8.00]	4.00 [2.00;8.00]	4.00 [2.00;8.00]	<0.001	<0.001
**Regional lymphnodes positive. n (%)**					<0.001	0.023
≤ 4	5497 (41.4%)	333 (42.5%)	4951 (42.3%)	227 (41.3%)		
> 4	5538 (41.8%)	291 (37.2%)	4457 (38.1%)	212 (38.5%)		
No	2227 (16.8%)	159 (20.3%)	2298 (19.6%)	111 (20.2%)		
**Rate of regional lymphnodes positive. median [IQR]**	0.29 [0.14;0.57]	0.24 [0.11;0.48]	0.33 [0.15;0.62]	0.33 [0.13;0.67]	<0.001	<0.001
**Rate of regional lymphnodes positive. n (%)**					<0.001	<0.001
≤ 31%	5701 (43.0%)	369 (47.1%)	4486 (38.3%)	209 (38.0%)		
> 31%	5334 (40.2%)	255 (32.6%)	4922 (42.0%)	230 (41.8%)		
No	2227 (16.8%)	159 (20.3%)	2298 (19.6%)	111 (20.2%)		
**Surgery for distant metastasis organ and lymphnode site. n (%)**					.	0.734
No	9629 (72.6%)	551 (70.4%)	8591 (73.4%)	369 (67.1%)		
Yes	3616 (27.3%)	231 (29.5%)	3094 (26.4%)	180 (32.7%)		
Missing	17 (0.13%)	1 (0.13%)	21 (0.18%)	1 (0.18%)		
**Radiation therapy. n (%)**					0.193	0.05
No/Missing	12874 (97.1%)	763 (97.4%)	11314 (96.7%)	531 (96.5%)		
Yes	388 (2.93%)	20 (2.55%)	392 (3.35%)	19 (3.45%)		
**Chemotherapy therapy. n (%)**					<0.001	<0.001
No/Missing	5111 (38.5%)	299 (38.2%)	3970 (33.9%)	185 (33.6%)		
Yes	8151 (61.5%)	484 (61.8%)	7736 (66.1%)	365 (66.4%)		

IQR: Interquartile range; CEA: Carcino-embryonic antigen
